# Sortilin is associated with breast cancer aggressiveness and contributes to tumor cell adhesion and invasion

**DOI:** 10.18632/oncotarget.3401

**Published:** 2015-03-18

**Authors:** Séverine Roselli, Jay Pundavela, Yohann Demont, Sam Faulkner, Sheridan Keene, John Attia, Chen Chen Jiang, Xu Dong Zhang, Marjorie M. Walker, Hubert Hondermarck

**Affiliations:** ^1^ School of Biomedical Sciences & Pharmacy, Faculty of Health and Medicine, University of Newcastle, Callaghan NSW 2308, Australia; ^2^ Hunter Medical Research Institute, University of Newcastle, New Lambton NSW 2305, Australia; ^3^ INSERM U908, IFR-147, Universite Lille 1, Villeneuve d'Ascq 59655, France; ^4^ School of Public Health & Medicine, Faculty of Health and Medicine, University of Newcastle, Callaghan NSW 2308, Australia; ^5^ INSERM U1138, Equipe 11, Centre de Recherche des Cordeliers, 15 rue de l'Ecole de Medecine, 75006 Paris, France

**Keywords:** breast cancer, sortilin, protein expression, cell adhesion, cell invasion

## Abstract

The neuronal membrane protein sortilin has been reported in a few cancer cell lines, but its expression and impact in human tumors is unclear. In this study, sortilin was analyzed by immunohistochemistry in a series of 318 clinically annotated breast cancers and 53 normal breast tissues. Sortilin was detected in epithelial cells, with increased levels in cancers, as compared to normal tissues (*p* = 0.0088). It was found in 79% of invasive ductal carcinomas and 54% of invasive lobular carcinomas (*p* < 0.0001). There was an association between sortilin expression and lymph node involvement (*p* = 0.0093), suggesting a relationship with metastatic potential. In cell culture, sortilin levels were higher in cancer cell lines compared to non-tumorigenic breast epithelial cells and siRNA knockdown of sortilin inhibited cancer cell adhesion, while proliferation and apoptosis were not affected. Breast cancer cell migration and invasion were also inhibited by sortilin knockdown, with a decrease in focal adhesion kinase and SRC phosphorylation. In conclusion, sortilin participates in breast tumor aggressiveness and may constitute a new therapeutic target against tumor cell invasion.

## INTRODUCTION

The expression of nervous system related proteins in cancer is an intriguing feature of several carcinomas that probably stems from the shared developmental origin of neurons and epithelial cells, which both derive from the neuroepithelial layer of the embryo. Neurotrophic growth factors [1], neuronal guidance molecules [2] or receptors for neurotransmitters [3] are expressed in tumors and, similarly to their role in the nervous system, may contribute to the plasticity of cancer cells.

Sortilin is a neuronal type-1 membrane protein, encoded by the *SORT1* gene, that belongs to the Vacuolar Protein Sorting 10 protein (VPS10P) family of receptors and is most abundantly expressed in both the central and peripheral nervous systems [4]. Sortilin is composed of a transmembrane segment and a short cytoplasmic tail, including motifs for interaction with cytosolic adaptor molecules. Initially described as the neurotensin receptor-3, sortilin is more generally involved in protein sorting and trafficking via a complex pattern whereby it shuttles between the cell surface and various intracellular compartments, directing target proteins to distinct destinations [5]. It is a common binding partner of tyrosine kinase receptors, G-protein coupled receptors and ion-channels, for which it facilitates ligand-induced signalling [6]. Sortilin has been identified as a co-receptor for neurotensin and pro-nerve growth factor (proNGF), and in the latter case acts in a complex with the neurotrophin receptor p75^NTR^ to induce neuron apoptosis [6, 7]. Further to its neuronal death-promoting activity, sortilin has also recently been identified as a receptor for apolipoprotein E and is a key factor in the catabolism of amyloid-β peptide in the brain [8]. Overall, sortilin is an essential regulator of neuronal viability and a potential therapeutic target in neurodegenerative diseases, but its role outside the nervous system, and particularly in cancer remains to be determined.

In non-neuronal tissues, sortilin expression has been reported in skeletal and heart muscles, adrenal gland, thyroid, lymphocyte B cells as well as keratinocytes and adipocytes [9–12]. A few cancer cell lines have been shown to express sortilin and are impacted by its disruption. In the HT29 colon cancer cells, sortilin participates in the control of growth promoting activity by brain-derived growth factor, through interacting with its tyrosine kinase receptor TrkB [13]. Additionally, sortilin mediates the release and transfer of exosomes in the A549 lung cancer cell line [14]. In prostate cancer cells, sortilin has been shown to regulate progranulin stimulatory activity of cancer cell growth [15]. In melanoma cell lines, sortilin is a co-receptor for pro-nerve growth factor (proNGF), and acts in cooperation with the neurotrophin receptor p75^NTR^ to promote cancer cell invasion [16]. Similarly, in breast cancer cell lines sortilin has been shown to participate in proNGF induced-cell invasion through cooperation with the tyrosine kinase receptor TrkA [17]. Together, data about the impact of sortilin in cancer are fragmentary, and as the expression of sortilin has never been reported in a cohort of human cancers, its clinicopathological significance in oncology is unclear.

In the present study, sortilin protein levels were analyzed by immunohistochemistry in a cohort of clinically annotated breast cancers and normal breast tissues. The expression of sortilin was found increased in breast cancer, particularly in ductal invasive carcinomas, and there was an association with lymph node invasion. In addition, decreasing sortilin protein level resulted in a diminished adhesion and invasion of breast cancer cells.

## RESULTS

### Sortilin protein expression in breast cancers

**Figure 1:**
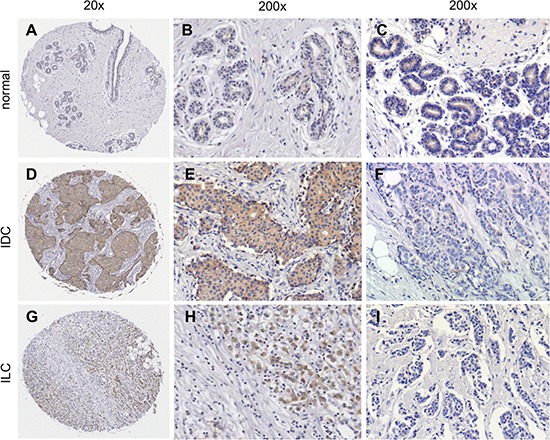
Immunohistological detection of sortilin in breast cancers The expression of sortilin was assessed by immunohistochemistry in a series of invasive breast cancers and normal adjacent tissues. Representative photos of sortilin immunolabeling are shown. **A.** Entire core and **B, C.** higher magnifications obtained for normal breast adjacent tissue; **D.** Entire core and **E.**) higher magnification obtained for an invasive ductal carcinoma (IDC) positive for sortilin; **F.** Sortilin negative IDC. **G.** Entire core and **H.** higher magnification obtained for an invasive lobular carcinoma (ILC) positive for sortilin; **I.** Sortilin negative ILC. Magnification (20x, 200x) is indicated.

**Figure 2:**
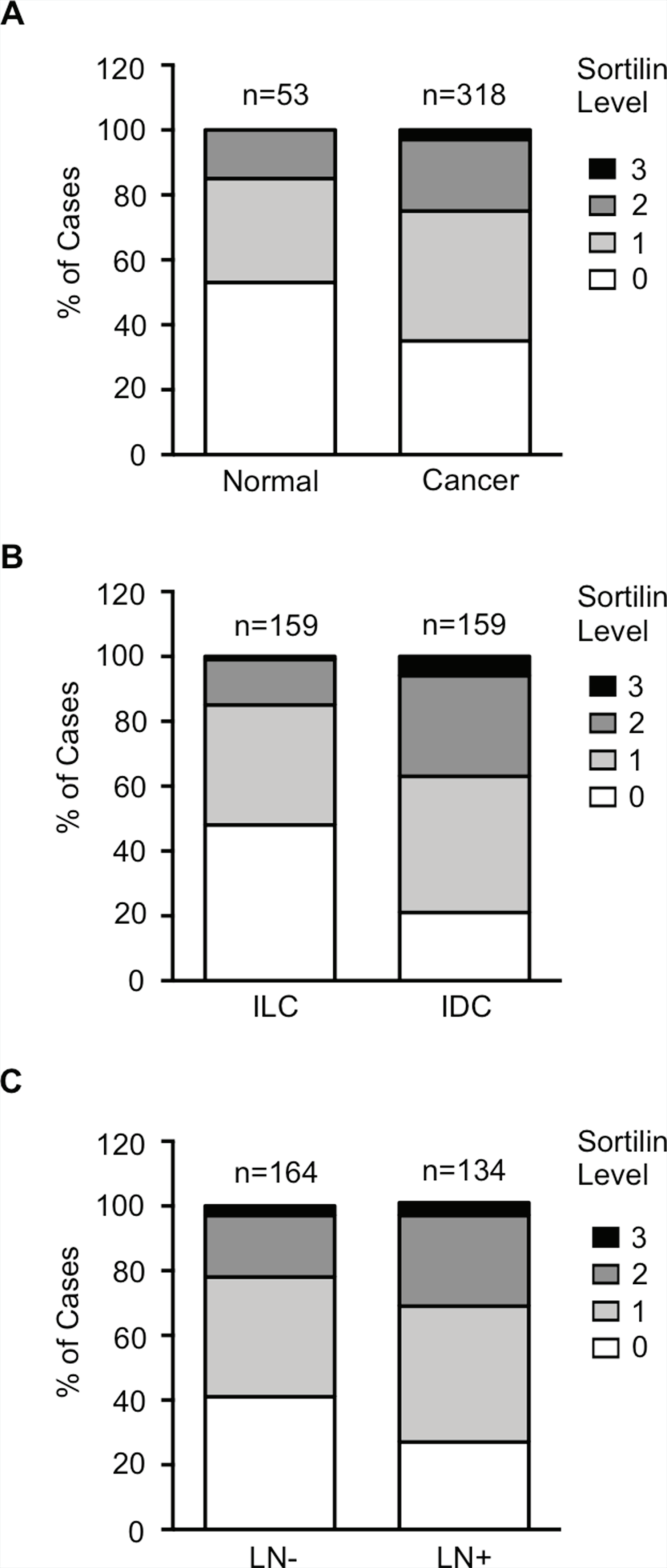
Frequency distribution of sortilin levels Sortilin levels (0 = no staining, 1 = low intensity staining, 2 = intermediate intensity staining, 3 = high intensity staining) were measured in breast cancers and normal breast tissues. **A.** Distribution in normal tissues versus breast tumors. **B.** Distribution in invasive lobular carcinomas (ILC) versus invasive ductal carcinomas (IDC). **C.** Distribution in lymph node negative (LN-) versus lymph node positive (LN+) cancers. Number of cases (n) is indicated. Statistical significance of the difference between groups are reported in Table 1.

Sortilin was analyzed by immunohistochemistry in a series of 318 clinically annotated breast cancers and 53 adjacent normal tissues. Sortilin expression was found only in epithelial cells of both normal and cancerous samples (Fig. 1). No labeling was observed in the stroma: fibroblasts, endothelial cells, adipocytes and extracellular matrix were all negative. The frequency distribution of sortilin levels (Fig. 2) showed that the majority of normal tissues had low levels of sortilin (staining intensity 0 and 1), while the proportion of cases with intermediate (staining intensity 2) and high (staining intensity 3) levels of sortilin increased in cancers and in particular in invasive ductal carcinomas (IDC) and lymph node positive tumors. There was a clear difference between sortilin positive and sortilin negative cases (Fig. 1) and among sortilin positive cases, the staining intensities were fairly homogeneous (mostly staining intensities 1 and 2). Therefore, the data were expressed in terms of sortilin positive *versus* sortilin negative cancer cases (Table 1). Analysis of relationships between sortilin expression and clinicopathological parameters revealed sortilin expression in 66% of breast cancers compared to 47% of adjacent normal tissues (*p* = 0.0088). A difference in expression between invasive ductal carcinomas (IDC) and invasive lobular carcinomas (ILC) was observed: 79% of IDC were positive for sortilin as compared to 54% of ILC (*p* < 0.0001). No significant association of sortilin expression was observed with tumor size, grade, patient age, ER and PR, and molecular subtypes of breast cancer (luminal A and B, HER2+, triple negative). Sortilin was expressed in 59% of triple negative breast cancers. In addition, there was a trend toward more tumors expressing sortilin among HER2-positive tumors (77%) than among HER2-negative tumors (63%) but the *p*-value was limited (*p* = 0.0349). A significant association was found between sortilin expression and lymph node invasion. Sortilin was expressed in 60% of lymph node negative cancers *versus* 75% of lymph node positive cancers (*p* = 0.0093), suggesting a positive relationship between sortilin expression and the metastatic potential. In Log-Linear modeling, two-way analyses confirmed the association, adjusted for all other variables, of sortilin with histological type (ductal vs. lobular invasive carcinomas, *p* = 0.002) and lymph node invasion (*OR* = 1.55 for lymph node positivity, *p* = 0.096).

**Table 1:** Association between sortilin expression and clinicopathological parameters in breast cancer

	Sortilin negative	Sortilin positive	*p*-value
**Normal vs cancer**			
Normal (*n* = 53)	28 (53%)	25 (47%)	0.0088
Cancer (*n* = 318)	107 (34%)	211 (66%)	
**Pathological type**			
IDC (*n* = 159)	34 (21%)	125 (79%)	<0.0001^a^
ILC (*n* = 159)	73 (46%)	86 (54%)	
**Age (years)**			
<50 (*n* = 171)	54 (32%)	117 (68%)	0.4075
≥50 (*n* = 147)	53 (36%)	94 (64%)	
**Tumor size**			
T1 (*n* = 25)	11 (44%)	14 (56%)	0.0951
T2 (*n* = 228)	75 (33%)	153 (67%)	
T3 (*n* = 31)	15 (48%)	16 (52%)	
T4 (*n* = 29)	6 (26%)	23 (74%)	
**Lymph node status**			
LN− (*n* = 164)	65 (40%)	99 (60%)	0.0093^a^
LN+ (*n* = 134)	34 (25%)	100 (75%)	
**HER2**			
HER2− (*n* = 252)	92 (37%)	160 (63%)	0.0349
HER2+ (*n* = 66)	15 (23%)	51 (77%)	
**Estrogen receptor**			
ER− (*n* = 182)	67 (37%)	115 (63%)	0.1670
ER+ (*n* = 136)	40 (29%)	96 (71%)	
**Progesterone receptor**			
PR− (*n* = 208)	75 (36%)	133 (64%)	0.2111
PR+ (*n* = 110)	32 (29%)	78 (71%)	
**Breast cancer subtypes**			
luminal A (*n* = 129)	44 (34%)	85 (66%)	0.1329
luminal B (*n* = 33)	9 (27%)	24 (73%)	
HER2 (*n* = 33)	7 (21%)	26 (79%)	
TNBC (*n* = 122)	50 (41%)	72 (59%)	

Abbreviations: IDC = Invasive ductal carcinoma; ILC = Invasive lobular carcinomas; HER2 = Human epidermal growth factor receptor 2; ER = estrogen receptor; PR = progesterone receptor; TNBC = Triple negative breast cancer.

Chi-square test was used to test statistical association. Statistically significant *P*-values (*p* < 0.05) are shown in bold.

a The association with histological type and lymph node invasion was confirmed by two-way Log-Linear analysis, but not the association with HER2+

### Sortilin expression in breast cancer cell lines

**Figure 3:**
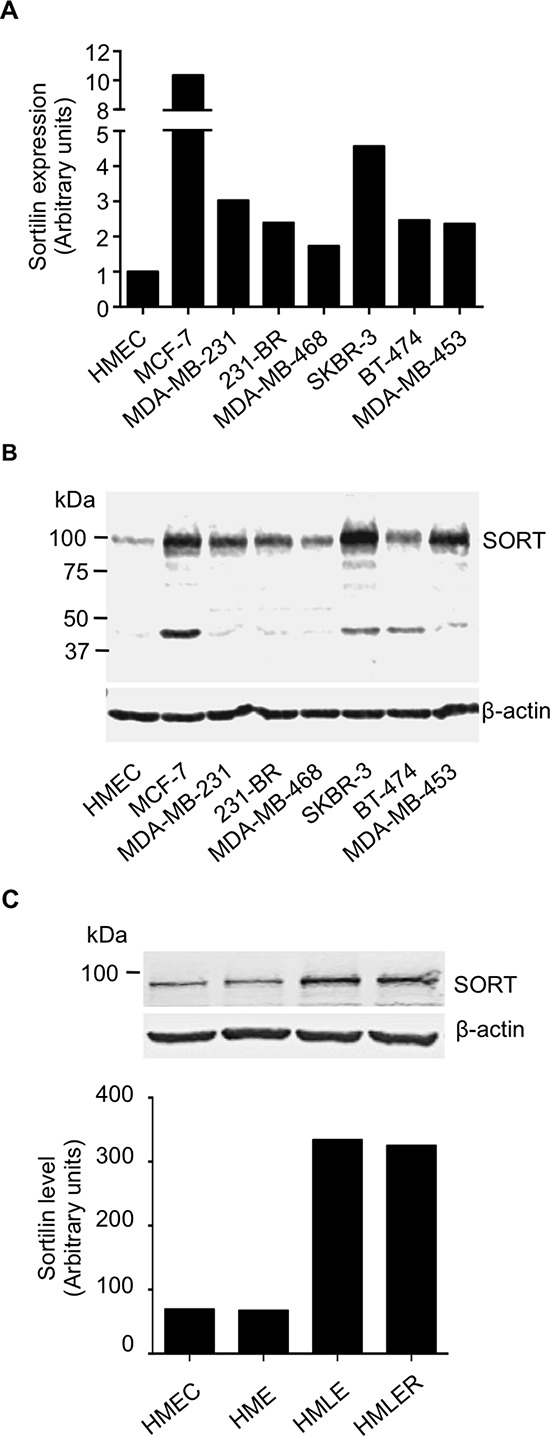
Expression of sortilin in breast cancer cell lines **A.** Quantitative RT-PCR analysis of sortilin gene expression in a range of breast cancer cell lines. Human mammary epithelial cells (HMEC) and the breast cancer cell lines MCF-7, MDA-MB-231 and their brain metastatic derivatives 231-BR, SKBR-3, MDA-MB-468, BT-474 and MDA-MB-453 were analyzed. Normalization was performed using β actin and the value obtained for HMEC was considered as 1. **B.** Western-blotting detection of sortilin in the same breast cancer cell lines. A band at about 100 kDa, the expected molecular weight of sortilin, was observed in all cell lines. In MCF-7, SKBR3 and BT474 cells, an additional band at 50 kDa was also detected. **C.** Sortilin was detected in the HMEC derivatives model of breast tumorigenic progression. The intensity of the sortilin band was higher in the tumorigenic HMLE and HMLER cells compared to the precancerous HME and the normal non-transformed HMEC.

A series of normal, immortalized and cancerous breast epithelial cells was analyzed for sortilin expression by RT-PCR and Western-blotting (Fig. 3). qRT-PCR analysis showed varying levels of sortilin mRNA in normal and cancer cell lines (Fig. 3A). All breast cancer cell lines expressed more mRNA for sortilin than the normal breast epithelial cells (HMEC). In Western-blotting, a band at about 100 kDa, which corresponds to the expected migration of sortilin, was observed in all tested cells (Fig. 3B). In MCF-7, SKBR-3 and BT-474 cells, an additional minor band at 50 kDa was also detected. This additional band may represent a degraded form of sortilin, which requires further characterization. Overall, there was more sortilin in cancer cell lines than in the normal HMEC. In the HMEC-derivatives model of breast carcinogenesis [18], there was an increase of sortilin in the tumorigenic HMLE and HMLER as compared to the normal HMEC and the transformed but non-tumorigenic HME (Fig. 3C) (the entire blot is shown in Supplemental Data).

### Impact of sortilin inhibition on breast cancer cell phenotype

**Figure 4:**
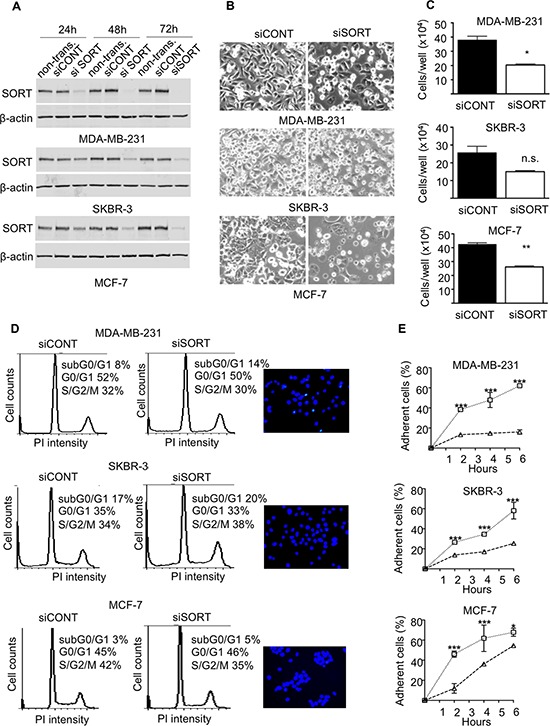
Impact of sortilin knockdown on proliferation, survival and adhesion of breast cancer cells **A.** SiRNA against Sortilin (siSORT) and universal negative control siRNA (siCONT) were transfected in MDA-MB-231, MCF-7 and SKBR-3 breast cancer cells, and the impact on the level of sortilin was measured by Western-blotting 24, 48 and 72 h after transfection. Non-transfected cells (non transf.) were also analyzed. **B.** Microscopic observation of breast cancer cells 72 h after transfection with siSORT and siCONT. **C.** Counting of breast cancer cells 72 h after transfection with siSORT versus siCONT. The histograms represent the mean number of cells per well. **D.** Flow cytometry analysis of breast cancer cells 72 h after transfection with siSORT or siCONT. The percentage of cells in SG2M, G0/G1 and subG0/G1 is indicated. For each cell line, a picture of Hoechst staining observed in siSORT is shown. **E.** Impact of siRNA against sortilin on breast cancer cell adhesion. Breast cancer cells were transfected with siRNA and were seeded in culture dishes. 48 h latter, number of attached cells was counted at the indicated times after seeding. Results are expressed, as percentage of adherent cells. For panel C and E, error bars represent SD. *p < 0.05, **p < 0.01, ***p < 0.001, n.s. not significant, for the difference between siCONT and siSORT.

**Figure 5:**
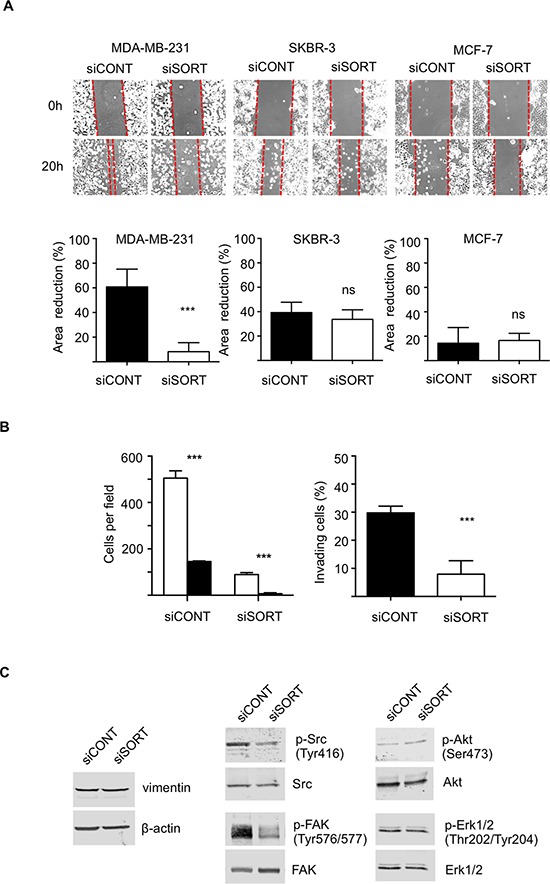
Impact of sortilin knockdown on migration and invasion of breast cancer cells **A.** Scratch assay. Breast cancer cells (MDA-MB-231, SKBR-3 and MCF-7) were transfected with siRNA against sortilin (siSORT) and universal negative control siRNA (siCONT). Scratching of the cell layer was performed 48 h after transfection and reduction in gap area was measured over 6 h. SiSORT inhibited migration only in MDA-MB-231 cells. **B.** Transwell invasion assay of MDA-MB-231 cells. Transwell assays were set up 48 h after siRNA transfection and cells were allowed to invade for 48 h. To take under account a potential impact of cell adhesion on the assay, cells were counted on both sides of the Transwell filter. Left panel, white columns represent the number of cells on the upper side of the filter, and the black columns the number of cells on the down side. Right panel, the percentage of invading cells in siSORT *versus* siCONT is represented. **C.** Western-blot detection of vimentin and activation of SRC, FAK, Akt and Erk1/2, 72 h after transfection with siSORT versus siCONT. Antibodies against vimentin, β-actin, SRC, phospho-SRC (Tyr416), FAK, phospho-FAK (Tyr576/577), Akt, phosphor-Akt (Ser473), Erk1/2, phosphor-Erk1/2 were used. For panel A and B, error bars represent SD. *p < 0.05, **p < 0.01, ***p < 0.001, n.s. not significant, for the difference between siCONT and siSORT.

The functional analysis was performed on the highly invasive and triple negative MDA-MB-231 breast cancer cell line, the HER2 overexpressing SKBR-3, and the luminal A type MCF-7 cells. Breast cancer cell lines were transfected with siRNA against sortilin *versus* control siRNA and the impact on cell growth, survival, adhesion, migration and invasion was measured. The efficacy of siRNA was assessed by Western-blotting at 24, 48 and 72 h after transfection (Fig. 4A). In MDA-MB-231 cells, a strong decrease in sortilin protein was observed from 24 h and was maintained after 72 h. In SKBR-3 and MCF-7 cells, the inhibition was complete only at 48 h, but was also maintained at 72 h. Microscopic observation 72 h after transfection (Fig. 4B) suggested that there were fewer cells in siRNA sortilin than in control siRNA, with a lower attachment (higher proportion of round cells). The decrease in cell number was confirmed by cell counting (Fig. 4C). This has prompted us to analyze cell cycle and apoptosis. Flow cytometry after propidium iodide incorporation (Fig. 4D) indicated no change in the proportion of cells in each phase of the cell cycle (G1/G0, S, G2M) between the siRNA sortilin and the siRNA control conditions. This demonstrated that the sortilin siRNA had no impact on cell proliferation. In addition there was also no change in subG0/G1, suggesting that siRNA against sortilin did not induce cell death. This was confirmed by Hoechst staining (Fig. 4D), as no particular nuclei fragmentation or condensation could be observed in the anti-sortilin siRNA condition. About 5% of apoptosis could be observed for all cell lines with or without anti-sortilin siRNA. Therefore, the decrease in cell number observed after sortilin siRNA transfection was not due to a decrease in cell proliferation or an increase in cell death. This has prompted us to test the impact of the sortilin siRNA on cell adhesion. Interestingly, breast cancer cell adhesion was affected by sortilin siRNA knockdown (Fig. 4E). SiRNA against sortilin resulted in 30% inhibition of MCF-7 cell adhesion, as measured 20 h after cell seeding. In SKBR3, the inhibition of cell adhesion was ~50% and it reached ~80% in MDA-MB-231 cells. These results indicated that sortilin is involved in breast cancer cell adhesion. We then investigated the impact of sortilin knockdown on breast cancer cell migration and invasion (Fig. 5). In wound healing assay, anti-sortilin siRNA inhibited the migration of MDA-MB-231 cells (Fig. 5A). In contrast the migration of MCF-7 and SKBR-3 cells was not affected (Fig. 5A). We then looked for the invasive property of MDA-MB-231 in Transwell assays. The invasion of MDA-MB-231 cells was significantly inhibited by anti-sortilin siRNA (Fig. 5B). To take under account the potential impact of the inhibition of cell adhesion on the invasion of MDA-MB-231 cells, we counted the number of cells attached in both the upper part and the down-side of the Transwell filters (Fig. 5B left panel). We then expressed the percentage (%) of invading cells, as compared to attached cells (Fig. 5B right panel). The results show that the invasion of cancer cells that had attached was inhibited and therefore, siRNA against sortilin had a direct inhibitory effect on MDA-MB-231 cell invasion. We have then explored the level of activation of various cell invasion-related signaling pathways (Fig. 5C) (the entire blots are shown in Supplemental Data). Western-blotting experiments revealed that the level of vimentin was not affected by siRNA against sortilin, indicating that the EMT (epithelial-mesenchymal transition) phenotype of MDA-MB-231 was not altered. Akt and Erk1/2 phosphorylation was also not modified, but in contrast, the activation of SRC and focal adhesion kinase (FAK) was inhibited by anti-sortilin siRNA. Therefore, the sortilin knockdown-induced inhibition of MDA-MB-231 breast cancer cell invasion involves a decrease in SRC/FAK signaling pathways.

## DISCUSSION

This study is the first to report sortilin expression in a series of human tumors. The results highlight an increase in sortilin protein level in breast cancer cells, particularly in invasive ductal carcinomas, as well as an association between sortilin and lymph node invasion. Furthermore, the *in vitro* data point to a participation of sortilin in adhesion and invasion of breast cancer cells.

In terms of gene expression, sortilin mRNA abundance has not been reported to be linked to a particular molecular subtype of breast cancer or clinicopathological parameter. Data mining, using cBioportal [19] of The Cancer Genome Atlas (TCGA) database [20], which contains 1062 samples of invasive breast carcinomas, indicated that sortilin is altered in 7.2% of breast tumors with 5 cases of amplification, 2 homozygous deletions, 4 mutations, 59 mRNA up regulations, and 8 mRNA down regulations (data not shown). The 59/1062 cases of mRNA amplifications represented 5.5% of all breast cancer cases. In addition, using the Gene Expression-Based Outcome for Breast cancer Online (GOBO) [21] with datasets GSE1456, 3494, 7390, representing a total of 737 breast cancers, no relationship was found between sortilin mRNA abundance and clinicopathological parameters (molecular subtypes, lymph node invasion, ER, PR, HER2). Initial studies in yeast comparing mRNA *versus* protein levels have suggested a correlation of ~50% between mRNA and protein levels. In humans, global transcriptomic and proteomic analyses have shown that an estimated 30%–60% of changes in protein levels can be explained by corresponding variations in mRNA [23, 24]. In addition, a recent proteogenomics investigation in colorectal cancer [25] has revealed that mRNA abundance does not reliably predict protein abundance differences between tumors. This emphasizes the importance to analyse the protein level, as gene expression data may not reflect the abundance of the protein effectors in tumors.

In the present study, sortilin protein was found in a higher proportion of IDC than ILC. IDC represent the majority of breast cancers (~80%) and are generally more aggressive than ILC [26]. Sortilin expression was also detected across the molecular subtypes of breast tumors (luminal A, luminal B, HER2+ and triple negative/basal) with no significant difference. Interestingly, triple negative breast cancers, which do not express oestrogen receptor, progesterone receptor and the tyrosine kinase receptor HER2, were found to be positive for sortilin in 59% of cases. At this stage, triple negative breast cancers are characterized by what they don't express and they are the only molecular subtype of breast cancers for which there is no targeted therapies [27, 28]. As a consequence, triple negative tumors have a particularly poor prognosis with a higher propensity to metastasize. Our data suggest that sortilin could potentially be targeted in breast cancer, particularly in the aggressive and difficult to treat triple negative tumors.

The increased level of sortilin protein in breast cancers, alongside the association with lymph node invasion, has prompted us to look at the impact of sortilin inhibition in breast cancer cells. Our data indicated that decreasing the level of sortilin diminished breast cancer cell adhesion, while having no effect on cell proliferation and survival. Interestingly, soluble forms of sortilin have already been implicated in cell adhesion. In the colorectal cancer cell line HT29, recent studies have shown that soluble sortilin can regulate FAK-dependent activation of the PI3 kinase pathway [29] and that soluble sortilin impairs cell to cell cohesion [30]. In breast cancer cells, we have not detected any soluble forms of sortilin (data not shown), and the molecular mechanism involved in the inhibition of cell adhesion/invasion remains to be determined. Our study also shows that sortilin is involved in breast cancer cell invasion as knockdown of sortilin in the highly invasive MDA-MB-231 cells was found to inhibit cell invasion. The process of cancer cell invasion requires not only cell migration, but also digestion of the extracellular matrix, and changes in cell adhesion are closely associated to the metastatic process [31]. Circulating cancer cells have to attach to the endothelial barrier to establish new tumoral niches and thus remodelling of cell adhesion and invasion is a hallmark of metastatic cells. The kinases SRC and FAK are generally involved in cancer cell adhesion and invasion, including in breast cancer cells [32]. Activation of SRC and FAK can be initiated by integrins and various tyrosine kinase receptors, and we show here that sortilin knockdown resulted in a decreased activation of these kinases. On the other hand, Akt and Erk1/2 were not affected, showing that sortilin inhibition has a targeted effect on cell invasion-related signaling. Further experiments are necessary to precisely define the cellular proteins directly targeted by sortilin in breast cancer cells. It has previously been shown that sortilin acts as a co-receptor for proNGF and is necessary to induce the activation of the tyrosine kinase receptor TrkA [17]. However, in the present study, proNGF was not added to the culture media and therefore, our data show that the impact of inhibiting sortilin on breast cancer cells goes beyond the regulation of proNGF activity. Although the molecular mechanism of action of sortilin in breast cancer cells, and in particular its direct interacting partners, remain to be elucidated, our data suggest sortilin, as a new potential therapeutic target in breast cancer.

In a broader perspective, it is worth noting that sortilin is also a nociceptor involved in the transmission of pain feeling by sensory neurons [33], and therefore, targeting sortilin in oncology could also inhibit cancer pain. To date, there is no available drug against sortilin, however the synthesis of a first small molecule potentially capable of inhibiting sortilin has recently been described [34] and further developments could lead to clinically relevant inhibitors [35]. As sortilin can induce neuronal apoptosis [6, 7], future sortilin inhibitors are anticipated to promote neuron survival and be of potential value for the treatment of neurodegenerative disease. Our study suggests that the inhibition of sortilin could also potentially be used in oncology to inhibit cancer cell invasion. In any case, the value of sortilin, as a potential target, in breast cancer and in other forms of cancer, warrants further consideration.

## MATERIALS AND METHODS

### Tumor microarrays

High-density tumor microarrays (TMA) of breast cancer biopsies and normal adjacent tissues were obtained from US Biomax Inc (Rockville, USA). These included 158 invasive ductal carcinomas, 159 invasive lobular carcinomas, and 53 normal adjacent tissues (TMAs Catalogue number BR1921 and BR1921a). Histopathological subtypes were reviewed by a pathologist (MMW). Clinical annotations included age, lymph node status, estrogen receptor (ER), progesterone receptor (PR) and human epidermal growth factor receptor 2 (HER2) status. This study was approved by the Human Research Ethics Committee of the University of Newcastle Australia.

### Immunohistochemistry

After deparaffinization and rehydration, the TMAs were treated for immunohistochemistry as previously described [36]. Primary antibodies were rabbit polyclonal anti-sortilin (Cat ANT-009, Alomone Labs, Jerusalem, Israel) and non-immune rabbit IgG control (Alpha Diagnostic, San Antonio, USA) at 0.8 μg/mL. Sortilin labeling was scored by two independent observers including a pathologist, on a scale ranging from 0 to 3, as follows: 0 (no staining), 1 (low intensity staining), 2 (moderate staining), and 3 (strong staining).

### Analysis of associations between sortilin expression and clinicopathological parameters

For the purpose of the analysis, because the labeling was homogeneous among sortilin positive cases, the scores were then grouped into two categories: sortilin negative (score 0) and sortilin positive (scores 1, 2, and 3). Simple unadjusted associations between sortilin and other pathological variables were performed using a chi-squared test. We used log-linear models to adjust the various bivariate associations for other potential confounders. The log linear models provided a Chi-squared test adjusted for all other variables; these included cancer type (lobular vs. ductal), lymph node involvement (yes/no), estrogen receptor positivity (yes/no), progesterone receptor positivity (yes/no), HER2+ (yes/no). The model was specified as a Poisson generalized linear model with a log-link function. Using hierarchical nesting of models we looked at all 3-way then 2-way interactions involving sortilin. Goodness of fit was tested using G2 Chi-squared statistics, as well as AIC and BIC. These models were fitted using SAS (SAS Institute, North Carolina, USA).

### Cell culture

Breast cancer cells MCF-7, MDA-MB-231, SKBR-3, MDA-MB-468, MDA-MB-453, BT-474 were obtained from the American Type Culture Collection (ATCC, Manassas, USA). The brain metastatic 231-BR cell line was a generous gift from Dr Barbara Steeg (Bethesda, USA). HMEC (human mammary epithelial cells), as well as their derivatives (HME, HMLE, HMLER), were obtained from Dr Robert Weinberg (Boston, USA). Individual cell line authentication was performed after DNA extraction (Promega kit, catalogue number A1120) and using the GenePrint 10 PCR amplification kit (Promega catalogue number B9510). All cancer and non-tumorigenic cell lines were maintained in RPMI-1640 with 10% (v/v) fetal calf serum (FCS) (JRH Biosciences, St. Louis, USA) and 2 mM L-glutamine in a humidified incubator at 37°C with 5% (v/v) CO_2_.

### Transfection with siRNA

Cells were transfected with siRNA using lipofectamine RNAiMAX (Life Technologies) according to manufacturer's recommendations. Cells were seeded in 6-well plates and transfected 24 h later with siRNA against sortilin (siSORT CUCUGCUGUUAACACCACC[dT][dT] or a siRNA control sequence commercially available from Sigma (MISSION^®^ siRNA Universal negative control #1). The efficiency of sortilin knockdown was assessed by Western-blotting using anti-sortilin antibody (ANT009, Alomone Labs, Israel). Actin detection (Cat antibody A2066, Sigma-Aldrich, St. Louis, USA) was used, as equiloading control.

### Western-blotting

Western-blotting experiments were performed, as previously described [36], with anti-Sortilin (1:500 dilution; Cat ANT-009, Alomone Labs, Jerusalem, Israel) and mouse anti-β-actin (1:5000 dilution; Sigma-Aldrich, St. Louis, USA). Antibodies from Cell Signaling Technology (USA) were also used for SRC (cat 2100), phosphoSRC (Tyr416, cat 2101), FAK (cat 1009), phosphoFAK (Tyr576/577, cat 3281), Erk1/2 (cat 9107), phosphoErk1/2 (Thr202/Tyr204, cat 4370), Akt (cat 9272), phosphoAkt (Ser473, cat 9271), vimentin (cat 5741).

### Quantitative reverse transcription-PCR (qRT-PCR)

Total RNA was isolated from breast cancer cell lines using the illustra RNAspin Mini RNA Isolation Kit (GE Healthcare Life Sciences, Little Chalfont, UK). Reverse transcription was performed with 1 μg of total RNA using the iScript cDNA Synthesis Kit (Bio-Rad Laboratories, Inc., Hercules, USA). Real-time PCR was performed using 2μl 1/10 cDNA using iTaq Universal SYBR Green Supermix (Bio-Rad Laboratories, Inc., Hercules, USA). Sortilin Primers were Quantitect Primer Assay QT00073318 (Qiagen, Venlo, Netherlands). The PCR was carried out in a ABI7500 Real-Time PCR System (Applied Biosystems, Thermo Scientific, Waltham, USA) using the following conditions, 95°C for 10 minutes, 40 cycles of 95°C for 15 seconds and 60°C for 60 seconds followed by a continuous Melt curve from 65°C to 95°C. Data analysis was performed using the ABI7500 Real-Time Software (Applied Biosystems, Thermo Scientific, Waltham, USA). Relative expression was obtained using the 2^−ΔΔCt^ method.

### Flow cytometry

Breast cancer cells (MDA-MB-231, SKBR-3, MCF-7) were collected by trypsinization after 72 h siRNA transfection, pooled with the saved growth media, and pelleted at 500 x g for 5 min. After PBS wash and counting, 10^6^ cells were gently resuspended in 400 μL of ice-cold PBS followed by addition of 800 μL ice cold 100% (v/v) ethanol in order to achieve fixation in 66% (v/v) ice cold ethanol at 4°C overnight. On the day of cell cycle analysis, the fixed cell samples in ethanol were equilibrated to room temperature, gently re-suspended and pelleted at 500 × g for 5 min followed by a PBS wash. Labeling was performed by addition of 500 μL of FxCycle Propidium iodide/RNase staining solution (Life Technologies, USA) to each sample and incubation for 15–30 min at room temperature in the dark. Cell cycle analysis was performed with a BD FACSCanto flow cytometer (Becton Dickinson, Sydney, Australia) and the data was analyzed using the WEASEL software (WEHI, Melbourne, Australia). The percentage of cells in the different phases of the cell cycle (G0/G1, S, G2/M) as well as the subG0/G1 (indicative of cell death) was determined.

### Hoechst staining

The proportion of cells in apoptosis was determined using Hoechst staining, as previously described [37].

### Adhesion Assay

Breast cancer cells were transfected with anti-sortilin or control siRNA as indicated above. After 48 h they were detached using trypsin free TrypLE dissociation solution (Invitrogen, Thermo Scientific, Waltham, USA), and seeded at 10^5^ cells/mL in 12-well cell Corning culture plates (Corning, USA). After 2, 4, and 6 h, adherent cells were counted under a phase contrast microscope. Adherent cells appeared flat and attached, while non-adherent cells were round and mobile. Counting was performed in 5 random fields per culture dish. The assay was done in triplicate.

### Migration assay

Breast cancer cells were seeded in 6-well plate (5 × 10^5^ cells per well) and transfected with anti-sortilin or control siRNA. After 48 h, the cell monolayer was scratched with a 200 μL pipette tip, rinsed three times with PBS and replaced with media containing 0.1% (v/v) FCS. The gap area that resulted from the scratch was monitored by taking pictures of three random areas using a phase contrast microscope (Zeiss) over 6 h post-scratch. Results are shown, as the percentage reduction of the gap area measured using ImageJ (NIH).

### Invasion assay

Cell invasion assays were performed in 12-well Boyden microchambers (Transwell^®^) with 8 μm pore size membranes. Transwells were first coated with 100 μL of starvation medium with 0.1% (v/v) FCS plus 40 μg of rat-tail collagen I for 1 h at 37°C. Cell loading was done with 100,000 siRNA transfected cells (48 h after transfection) in 400 μL starvation medium with 0.1% (v/v) FCS in the upper chamber whereas 1.6 mL starvation medium with 0.1% (v/v) FCS was placed in the lower chamber. After 20 h of incubation, the Transwell filters were rinsed with PBS and cells at the upper surface of the membrane were gently scraped and removed for counting. Cells having invaded to the down side of the membrane were fixed and stained with 0.1% (w/v) crystal violet before counting (10 fields per membrane) through an inverted microscope.

### Statistical analysis

In the cell growth, adhesion, migration and invasion assays, each condition was performed in triplicate and statistical analysis was conducted using GraphPad Prism 6. The results of cell growth, migration and invasion assays were compared using a *t*-test and cell adhesion over time was compared using repeated measure two-way ANOVA.

## SUPPLEMENTARY DATA


