# Supplementary material for: Sortilin is associated with breast cancer aggressiveness and contributes to tumor cell adhesion and invasion
Source: Oncotarget. 2015 Mar 18;6(12):10473–86. doi: 10.18632/oncotarget.3401 (PMC4496368; doi:10.18632/oncotarget.3401)
Supplement: Supplementary file 1 [file oncotarget-06-10473-s001.pdf]

SUPPLEMENTARY DATA

Fig. 3C

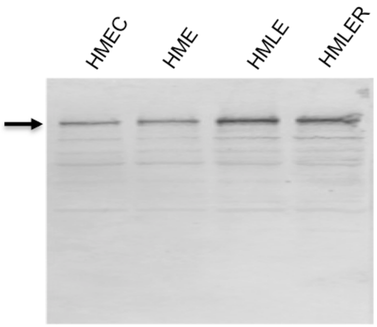

Fig. 5C

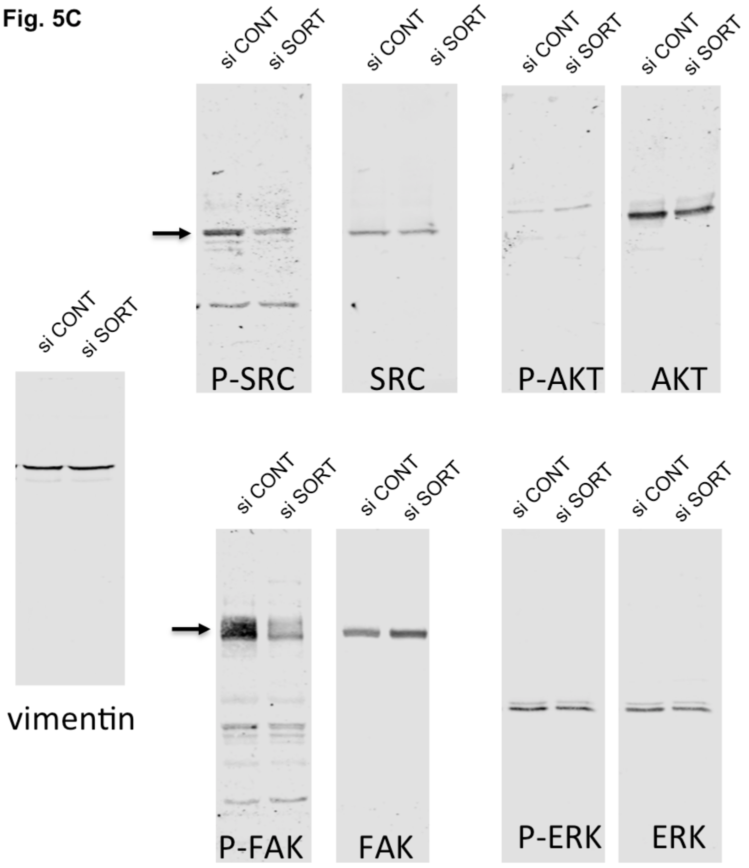

Supplementary Data S1: Entire blots corresponding to Fig. 3C and Fig. 5C.
